# Effects of Online Mental Health Classes on Mental Health and Stigma: a Controlled Before-After Study with 1-Month Follow-Up

**DOI:** 10.1007/s42399-022-01225-x

**Published:** 2022-07-27

**Authors:** Yasuhiro Kotera, Ann-Marie Edwards, Gulcan Garip, James Chircop, Muhammad Aledeh

**Affiliations:** 1 School of Health Sciences, University of Nottingham, Nottingham, UK; 2 College of Health, Psychology and Social Care, University of Derby, Derby, UK

**Keywords:** Mental health education, Stigma, Long-term plan, Controlled before-after study, Online education

## Abstract

Though the importance of mental health education has been emphasised, how learning about mental health helps the learners’ mental health remains to be evaluated. Accordingly, this study aimed to appraise the mental health effects of online mental health classes in a controlled before-after study with a 1-month follow-up. The Depression, Anxiety and Stress Scale-21 and Depression Stigma Scale were completed by 16 students in a mental health class and 12 in a non-mental health class. While there was no significant difference in depression, anxiety and stress, between groups (type of class) and within groups (assessment points), the levels of stigma were significantly lower in mental health students than non-mental health students at post-semester (*p* = .004). Findings illustrate temporal effectiveness of mental health classes on stigma; however, continuous education is needed to maintain the effects. Educators in mental health are recommended to design a long-term plan to support learners’ mental health.

## Introduction

As the awareness of mental health increases globally, the importance of mental health education has been emphasised [1]. Though the definition of “mental health” is still being debated, multidisciplinary international research identified its core concept as “the ability or capacity of a person to effectively deal with or change his/her environment” [2]. Likewise, Galderisi et al. proposed a new definition of mental health considering the negative emotions as part of a common human life, stating that “a dynamic state of internal equilibrium which enables individuals to use their abilities in harmony with universal values of society” [3]. Gaining the right knowledge about mental health, which is “mental health education”, can help people have less stigmatised views towards mental health problems and take appropriate actions including seeking help [4–7]. More recently, experiential components of mental health are embedded in many mental health education programmes such as mindfulness- and/or compassion-based activities [8]. Free online courses about mental health have been also developed such as Massive Open Online Courses (MOOCs) to offer knowledge and practical exercises to wider audience [9–11].

Negative impacts of learning about mental health have also been reported: Students learning about mental health symptoms such as depression became more depressed [12, 13]. This is similar to a more established notion of “medical student syndrome”, where medical students develop symptoms that relate to the diseases they are studying [14]. Although research about the mental health aspect of the medical student syndrome remains to be evaluated [15], a serious concern was raised [16]. Indeed, the medical students’ syndrome—medical students falsely relate themselves with a health condition that they are learning, impacting their health negatively [14]—may apply to the mental health contexts too. However, the “mental health students’ syndrome” in the online domain remains to be examined. Accordingly, this study aimed to evaluate the mental health impact of undertaking an online mental health class.

Mental health among university students is a cause for concern in many countries [17]. Students in the UK report high rates of mental health problems [18]. Likewise, students in the Czech Republic and Republic of Ireland also demonstrate poor mental health [19–21], for example, the majority of educators in more than 2000 business schools in the USA recognised that stress was a great concern among students. Turning to Asia, similar mental health concerns were identified in many countries such as Japan and Malaysia [17, 22]. Many students experience a transition in terms of living away from their families for the first time, increased academic work and social activities, and pressure to secure employment [23]. This has led to a greater demand for mental health education: More and more students undertake a mental health class today because of their own or family member’s lived experience with mental health difficulties [12]. While these classes are designed to increase knowledge and skills about mental health, whether these classes are helpful to their mental health remains uncertain. Indeed, recent studies [12, 13] reported its negative impact; however, little is known about online education.

Lastly, it is noteworthy that demand for online education has increased alongside the restrictions associated with the COVID-19 pandemic [24]. The demand is expected to increase in the coming years [25]. Online education offers greater access to more students [26] by using a variety of tools and systems to help students engage in learning remotely [27]. This feature was particularly of high value when restrictions were in place during the pandemic.

Taken together, the present study aimed to assess the mental health effects of students studying online mental health classes versus those studying non-mental health classes in a controlled before-after study with a 1-month follow-up.

## Methods

Participants were recruited via programme announcements by academics who were not a researcher of this study at the beginning of week 0, 1 week before a semester (September 2020). To participate, students had to be currently studying online and not undertaking any other classes: Students on a study break were excluded. Students log into their online learning platform and engage in a series of learning activities. Tutors offer feedback to students’ output from the activities.

Students who have agreed to participate were asked to complete the baseline assessment by the end of week 1. To blind the groups, they were introduced to the study that evaluates the mental health impact of undertaking an online class: The grouping was informed to the students after the study. In total, approximately 300 students were approached in week 0. Seventy-six students initially agreed to take part, of whom 28 completed the three assessments: 16 in a mental health class (11 females and 5 males; age 22–57, 41.50 ± 10.03 years; 6 psychology students, 5 nursing students and 5 counselling students) and 12 in a non-mental health class (12 females; age 33–47, 39.17 ± 4.73 years; 6 nursing students and 6 counselling students). The mental health classes included “psychopathology” and “working with people with distress and disorders” where acquiring knowledge and skills in mental health was the primary purpose of the class (confirmed with the class lead), whereas the non-mental health classes included “diabetes: a contemporary approach” and “skills in supervisory practice”. In the mental health classes, students learned how to diagnose a mental disorder using the Diagnostic and Statistical Manual of Mental Disorders, Fifth Edition (DSM-5) [28] or the International Statistical Classification of Diseases and Related Health Problems, 11th Revision (ICD-11) [29]; social model of mental illness [30]; stigma and shame associated with mental health problems; and mental health practitioners’ account on working with people with mental health problems (e.g. self-care).

All of these classes have been validated by internal and external expert panels of the university, ensuring that they meet the quality standards set by the UK’s Quality Assurance Agency for Higher Education (QAA) [31]. Moreover, the contents and teaching practices are reviewed every year considering feedback from students, staff, external examiners and professional bodies. Matters discussed are shared among these stakeholders regularly. The post-semester assessment took place for 2 weeks after the teaching weeks (weeks 11 and 12), and the follow-up assessment was conducted four weeks after (weeks 15 and 16). The assessment tool comprised the Depression, Anxiety and Stress Scale-21 (DASS-21) [32] and Depression Stigma Scale (DSS) [33], administered online. No compensation was offered for participation. Reason for withdrawal was not asked per ethical guidelines; however, no complaint was received.

Once data was screened for outliers, normalities, homogeneity of variances, and multicollinearity, a multiple analysis of variance (MANOVA) was conducted. Ethical approval was obtained from the university ethics committee.

## Results

All data were subjected to evaluation for significant differences in the mental health and stigma scores within data collection points (baseline, post-semester and a one-month follow-up) and between conditions (mental health classes and non-mental health classes) using a MANOVA. Partial eta squared was calculated as the effect size parameter using Cohen’s guidelines [34] (0.2 = small, 0.5 = medium, 0.8 = large).

**Fig. 1:**
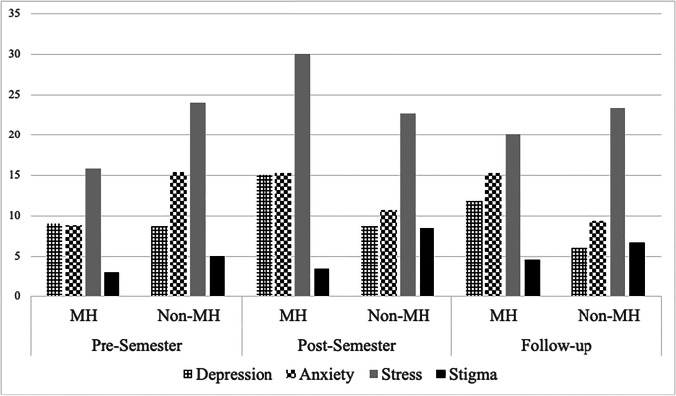
Online class effects on mental health and stigma at 3 time points. Significant difference in stigma between MH students and non-MH students at post-semester but not at 1-month follow-up

The interaction effect between the class type and the data collection points on the combined dependent variables was not statistically significant, *F*(8, 148) = 1.07, *p* = 0.39, Wilks’ Λ = 0.89, partial *η*^2^ = 0.06. There was no significant difference among assessment points on the combined dependent variables, *F*(4, 152) = 0.87, *p* = 0.55, Wilks’ Λ = 0.91, partial *η*^2^ = 0.05. However, the combined dependent variables were significantly different by the type of class, suggesting that at least one of the dependent variables differed significantly between the two groups at least one assessment point, *F*(4, 74) = 4.36, *p* = 0.003, Wilks’ Λ = 0.81, partial *η*^2^ = 0.19 (small effect size). Tukey post hoc pairwise comparisons identified that depression stigma score reported post-semester was significantly lower in mental health classes (3.31 ± 3.81) than non-mental health classes (8.50 ± 5.28) (95% CI 4.11–7.71), *F*(1, 77) = 8.88, *p* = 0.004, partial *η*^2^ = 0.10 (small effect size). Table 1 and Fig. 1 summarise our findings.

**Table 1:** Online class effects on mental health and stigma at 3 time points

	Pre-semester				Post-semester				1-month follow-up			
	Mental health classes		Non-mental health classes		Mental health classes		Non-mental health classes		Mental health classes		Non-mental health classes	
	*M*	*SD*	*M*	*SD*	*M*	*SD*	*M*	*SD*	*M*	*SD*	*M*	*SD*
Depression	9.00	11.55	8.67	13.52	15.00	16.94	8.67	8.15	11.75	15.56	6.00	9.57
Anxiety	8.75	11.24	15.33	16.43	15.25	14.91	10.67	9.24	15.25	20.14	9.33	10.97
Stress	15.75	13.66	24.00	13.86	30.00	23.46	22.67	21.26	20.00	22.29	23.33	16.26
Stigma	2.94	3.17	5.00	3.36	3.31*	3.81	8.50*	5.28	4.56	6.50	6.67	5.18

^*^Significant difference between the two

## Discussion

The present study assessed the mental health effects of students studying online mental health classes versus those studying non-mental health classes in a controlled before-after study with a 1-month follow-up. The main finding from this study was that students studying a mental health class had, on average, lower levels of stigma compared to those students in non-mental health classes at the end of the semester. However, the reduction in stigma for students in mental health classes was temporal and not maintained one month later. These findings suggest, without actively engaging with mental health content, stigma towards mental health returns to baseline (pre-semester) levels. Mental health education can be an important resource for reducing stigma; however, for the benefits to be maintained, continued exposure to mental health content, and training in related concepts, such as emotional intelligence [35, 36], may be needed. Thus, it is recommended that educators in mental health make longer-term plans for improving mental health, which go beyond the teaching of mental health in the classroom. Integrating mental health-related topics, such as psychological flexibility [37], to other areas of student life (e.g., mental health-related activities in halls of residence) may help to reduce and maintain lower levels of stigma towards mental health.

Limitations of this study need to be noted. First, the sample sizes were small. This exploratory study does not require power calculation [38]; however, our findings from small samples lacked generalisability. Second, self-report measures were used, indicating possible response biases [39]. Third, we did not consider the impact of COVID-19 on online education, and both student and staff wellbeing [27, 40]. Lastly, this study was conducted at one university. Studies with larger samples at multiple settings are needed.

## Conclusion

The awareness of mental health and the demand for online education have been increasing worldwide. This study reported how mental health online education affects students’ mental health. Online students in mental health classes demonstrated less stigma associated with mental health issues than online students in non-mental health classes after a semester. Our findings offer foundational understanding of the impact of online mental health education on learners’ mental health.

## Data Availability

The data that support the findings of this study are available from the corresponding author, Yasuhiro Kotera, upon reasonable request.

## Code Availability

N/A.
